# Automatic vs. manual curation of a multi-source chemical dictionary: the impact on text mining

**DOI:** 10.1186/1758-2946-2-3

**Published:** 2010-03-23

**Authors:** Kristina M Hettne, Antony J Williams, Erik M van Mulligen, Jos Kleinjans, Valery Tkachenko, Jan A Kors

**Affiliations:** 1 Department of Medical Informatics, Erasmus University Medical Center, Rotterdam, The Netherlands; 2 Department of Health Risk Analysis and Toxicology, Maastricht University, Maastricht, The Netherlands; 3 Royal Society of Chemistry, 904 Tamaras Circle, Wake Forest, NC-27587, USA

## Abstract

**Background:**

Previously, we developed a combined dictionary dubbed Chemlist for the identification of small molecules and drugs in text based on a number of publicly available databases and tested it on an annotated corpus. To achieve an acceptable recall and precision we used a number of automatic and semi-automatic processing steps together with disambiguation rules. However, it remained to be investigated which impact an extensive manual curation of a multi-source chemical dictionary would have on chemical term identification in text. ChemSpider is a chemical database that has undergone extensive manual curation aimed at establishing valid chemical name-to-structure relationships.

**Results:**

We acquired the component of ChemSpider containing only manually curated names and synonyms. Rule-based term filtering, semi-automatic manual curation, and disambiguation rules were applied. We tested the dictionary from ChemSpider on an annotated corpus and compared the results with those for the Chemlist dictionary. The ChemSpider dictionary of ca. 80 k names was only a 1/3 to a 1/4 the size of Chemlist at around 300 k. The ChemSpider dictionary had a precision of 0.43 and a recall of 0.19 before the application of filtering and disambiguation and a precision of 0.87 and a recall of 0.19 after filtering and disambiguation. The Chemlist dictionary had a precision of 0.20 and a recall of 0.47 before the application of filtering and disambiguation and a precision of 0.67 and a recall of 0.40 after filtering and disambiguation.

**Conclusions:**

We conclude the following: (1) The ChemSpider dictionary achieved the best precision but the Chemlist dictionary had a higher recall and the best F-score; (2) Rule-based filtering and disambiguation is necessary to achieve a high precision for both the automatically generated and the manually curated dictionary. ChemSpider is available as a web service at http://www.chemspider.com/ and the Chemlist dictionary is freely available as an XML file in Simple Knowledge Organization System format on the web at http://www.biosemantics.org/chemlist.

## Background

Finding chemical terms in free text is essential for text mining aimed at exploring how chemical structures link to biological processes [1]. However, the techniques behind current text mining applications have mainly focused on the ability of the system to correctly identify gene and protein names in text, while less effort has been spent on the correct identification of chemical names [2,3]. This is however about to change as more and more chemical resources are becoming freely available [4-6]. For example, resources such as DrugBank [7] and the Unified Medical Language System metathesaurus (UMLS) [8] have been applied for the identification of drug names in text [9,10] (for a recent review of literature mining in support of drug discovery see Agarwal and Searls [11]). Briefly, the challenges of chemical name identification differ from the ones in the genomics field in the sense that the exact placement of tokens such as commas, spaces, hyphens, and parentheses plays a much larger role. Chemical named entity recognition (NER) in general has been reviewed by Banville [1] and methods for confidence-based chemical NER have been evaluated by Corbett and Copestake [12].

In this paper we focus on the task of term *identification*, which goes beyond NER to also include term *mapping*, i.e. the linking of terms to reference data sources. In the case of chemicals, they can also be identified by a specific structure representation such as a connection table, an InChI string or a simplified molecular input line entry specification (SMILES). To achieve this, a dictionary with database links, or structures, is essential. Naturally, the usefulness of the dictionary approach depends on the coverage of terms in the dictionary for the particular domain and how well the terms are suited for natural language processing. Previously, we developed a combined dictionary named Chemlist for the identification of small molecules and drugs in text based on a number of publicly available databases and tested it on an annotated corpus [13]. To achieve an acceptable precision (0.67) and recall (0.40) we used a number of automatic and semi-automatic processing steps together with disambiguation rules. However, it remained to be investigated which impact an extensive manual curation of a multi-source chemical dictionary would have on chemical term identification in text. We expect that a higher precision can be reached with a manually curated dictionary.

Around 8% of the chemicals in Chemlist contain structure information in the form of InChI strings. It should be noted that we did not validate the correctness of the association between the chemical names and the chemical structures/compounds as that was not the focus of the work. The challenges of chemical NER are clearly not limited only to the identification and extraction of a particular chemical name but also the association of the chemical name with an appropriate chemical structure or compound. ChemSpider [14] is an online database of chemical compounds and associated data and was developed with the intention of building a structure-centric database for the chemistry community. The chemicals contained within the database are sourced from over 200 different data sources including chemical vendors, government databases, commercial databases, open notebook science projects, blogs and personal chemistry collections deposited by members of the community. During the process of integrating and associating data from various sources the ChemSpider development team has identified a multitude of issues in regards to the quality of chemical structure representations. These include varying levels of accuracy in stereochemistry, the mis-association of chemical names with chemical entity and a myriad of other issues whereby chemical names are associated with incorrect chemical structures. The challenge here is one of assertion - what is a "correct" chemical structure and who asserts that it has a specific representation? While the chemical structure of benzene can be represented as either a series of alternating single and double bonds or as in a Kekule form, the connection table of atoms and bonds as captured in an electronic format remains consistent. In terms of compounds of biological interest the structure representation for a particular drug is based on the collective wisdom of the company registering the compound, the patent representation and a multitude of databases containing associated information. The challenges of both conventions and assertions are taken into account when creating a validated dictionary of chemical names and associated structure representations.

As a result of the challenges associated with poor quality chemical name-structure relationships ChemSpider was developed to include a curation platform whereby chemists could participate directly in the validation of the relationships. A web-based interface to approve, delete and add chemical names to chemical entities was delivered and a multi-level curator role was established so that when members of the community made suggested changes to the relationships master curators would then further investigate and approve their work. ChemSpider was released to the community in March 2007 and many tens of thousands of curation actions have provided a highly curated dictionary.

The objective of this study is to determine the impact of manual curation of chemical name-structure relationships on the precision and recall of chemical term identification.

## Results

The ChemSpider dictionary was filtered according to a set of pre-processing steps and tested on an annotated corpus (see Methods for details on the pre-processing steps and the corpus). Before pre-processing, the ChemSpider dictionary contained 157,173 terms belonging to 84,065 entities and after pre-processing 160,898 terms belonging to 84,059 entities. The processed version of Chemlist contains 1,692,020 terms belonging to 278,577 entities. Dictionary term strings that matched the start and end positions of the chemical term strings in the corpus constituted true positives (TP), term strings that were not marked as chemical term strings in the corpus but still matched a dictionary term string were false positives (FP), and chemical term strings in the corpus that were not matched were false negatives (FN). Recall (R), precision (P), and F-score were computed in the usual way:

• Recall = TP/(TP+FN)

• Precision = TP/(TP+FP)

• F-score = (2*P*R)/(P+R)

Table 1 shows the effect of pre-processing and disambiguation on precision and recall for the dictionaries. It is clear that the pre-processing steps and the disambiguation rules have a strong positive influence on the precision of both dictionaries. The ChemSpider dictionary had higher precision (0.87) and lower recall (0.19) compared to the Chemlist dictionary (precision 0.67 and recall 0.40). The Chemlist dictionary had the highest F-score (0.50). A combination of both dictionaries showed changes of less than 1 percentage point in recall and precision values (results not shown). The combination was created by matching concepts on CAS numbers and/or InChI strings, resulting in a merged dictionary with 317, 275 concepts. The overlap between the dictionaries was calculated to 45,361 concepts.

**Table 1:** Precision (P), recall (R) and F-score (F) of the dictionaries on the annotated corpus.

Dictionary	Unprocessed			Filtered			Frequent terms correction			Disambiguation		
	**P**	**R**	**F**	**P**	**R**	**F**	**P**	**R**	**F**	**P**	**R**	**F**

ChemSpider	0.43	0.19	0.26	0.81	0.19	0.31	0.85	0.19	0.31	0.87	0.19	0.31
Chemlist	0.20	0.47	0.28	0.39	0.46	0.42	0.55	0.46	0.50	0.67	0.40	0.50

Overall, the recall was best for the TRIV class of entities (Table 2), with Chemlist as the best performing dictionary (recall 0.80). The PART class of entities had the lowest recall of all classes (0.00, ChemSpider dictionary). The PART class is however more relevant when the corpus is going to be used for machine learning purposes since parts of chemical names are not expected to be found in dictionaries. This class was therefore left out of the error analysis below.

**Table 2:** Recall values for the entity classes per dictionary.

Entity class	ChemSpider	Chemlist
IUPAC (391)	0.08	0.21
PART (92)	0.00	0.04
SUM (49)	0.25	0.29
TRIV (414)	0.45	0.80
ABB (161)	0.01	0.22
FAM (99)	0.02	0.19

IUPAC: multiword systematic names, PART: partial chemical names, SUM: sum formulas, TRIV: trivial names (including single word IUPAC names), ABB: abbreviations, FAM: chemical family names.

### Error analysis

We performed a manual error analysis for the dictionaries with disambiguation rules applied (see Methods). The major reason that entities were not found (i.e., were false negatives) was that they simply were not in the dictionaries (Table 3). For the Chemlist dictionary, this holds true for all classes except ABB for which most belong to the category "removed by disambiguation". The major source of false positives for both dictionaries was partial matches of longer chemical names (Table 4). Notably, ChemSpider only had one entity out of corpus scope and no entities that were non-chemicals.

**Table 3:** Error analysis of a random sample of max 25 false negatives from each class for ChemSpider (CS) and Chemlist (CL).

Error type	TRIV		SUM		IUPAC		FAM		ABB	
	**CS**	**CL**	**CS**	**CL**	**CS**	**CL**	**CS**	**CL**	**CS**	**CL**

Partial match	0	3	0	0	0	0	0	0	0	0
Annotation error	0	2	0	0	0	1	0	0	0	0
Not in dictionary	25	15	22	16	25	24	25	24	25	8
Removed by disambiguation	0	5	0	7	0	0	0	1	0	12
Removed by manual check of highly frequent terms	0	0	0	1	0	0	0	0	0	2
Tokenization error	0	0	3	1	0	0	0	0	0	3

**Table 4:** Error analysis of the false positives (percentage) for ChemSpider and Chemlist.

Error type	False positives	
	**ChemSpider**	**Chemlist**

Partial match	21 (64%)	96 (41%)
Annotation error	11 (33%)	29 (13%)
Out of corpus scope	1 (3%)	79 (34%)
Not a chemical	0	28 (12%)

## Discussion

The Chemlist dictionary had the highest recall and the best F-score, but a lower precision than the ChemSpider dictionary. The precision of 0.87 (at a recall of 0.19) for the ChemSpider dictionary is the best reported for a chemical dictionary on the corpus used in this study. From the analysis of the false positives it was obvious that the ChemSpider dictionary was less out of the scope of the corpus and contained less non-chemical names than Chemlist. As mentioned in previous work [13], the false positives in the categories *entity out of corpus scope *and *annotation error *might possibly be excluded from the analysis because these errors cannot be attributed to the dictionaries. When the false positives from these categories were excluded, Chemlist had a precision of 0.82 and ChemSpider a precision of 0.91. Worth noticing is that the precision for the manually curated dictionary from ChemSpider on the corpus without the use of the pre-processing steps was about half compared to the processed version. A reason for this might be that the dictionary was not curated with text-mining purposes in mind. For example, synonyms such as "As" for "Arsenic" might be correct but will give rise to many false positives when the dictionary is used for text mining. However, it should be noted that the ChemSpider team used their own curated dictionaries as the basis of their semantic markup approaches on the ChemMantis [15] platform. Their entity extraction approach accounted for direct identification of elements and included a list of stop words to allow for improved precision.

The recall for the Chemspider dictionary is substantially lower than that of Chemlist in all categories except for the SUM class. It is to be expected that the ChemSpider dictionary scores lower for the FAM class since ChemSpider is a structure-centered database, but the relatively low recall for the IUPAC, TRIV and ABB classes were surprising. We therefore performed a search in the online version of ChemSpider (August 8, 2009) for the false negatives in these classes. Indeed, an additional 3 of the random 25 IUPAC false negative, 20 of the 25 random TRIV false negatives, and 10 of the 25 random ABB false negatives were found in the online version of ChemSpider. These differences might be explained by the update speed of the online ChemSpider database as hundreds of thousands of chemical entities can be added within a week. As of September 2009 there are eight million chemical entities waiting to be deduplicated into the ChemSpider database and there has been an increase of almost 10% in the unique number of chemical entities since this manuscript was started. There are presently over 23 million unique chemicals in the database.

Since despite the increased volume of ChemSpider only three of the 25 random IUPAC false negatives were found in the online version of ChemSpider, we performed a structural evaluation of the remaining 22 false negatives. This deep analysis of the structures of the false negatives from the IUPAC class highlights three different issues: firstly the annotation of the corpus, secondly the sometimes inconsistent or incorrect way scientists write chemical names in articles, and thirdly the ChemSpider database coverage.

The annotation issues follow. Two chemicals were annotated as IUPAC in the corpus but did not respond to unique structures (e.g. hexa-acetyl was annotated as IUPAC in the sentence "...it formed a **hexa-acetyl **derivative..."). We argue that these chemicals should be annotated as PART instead. Two cases were not chemical names but internal abbreviations in the abstract (e.g (S)-(-)-3-PPP). We argue that these should belong to the ABB class instead. These four cases reflect the relatively low annotator agreement on the corpus (80%) [16]. One annotation error concerns two chemicals after each other that were annotated as one in the sentence "On interaction with anhydrous **potassium acetate 14-bromcarminomycinone **(III) yield 14-acetoxycarminomycinone (IV)". Five annotation "errors" were family names (e.g. 1-(carboxyalkyl)hydroxypyridinones). These cases are however not annotation errors according to the class definition of the FAMILY class in Kolarik et al. [16], where "Substances used as bases for building various derivatives and analogs were tagged as IUPAC, not as FAMILY (e.g. 1,4-dihydronaphthoquinones)", but they are not expected to be found in ChemSpider since ChemSpider focuses on single compounds.

The way chemical names are written in articles concern the following cases: two were too generic to correspond to unique structures (e.g. 5-O-tetradecanoyl-2,3-dideoxy-L-threo-hexono-1,4-lactone), and six were non-systematic names for which no structure could be drawn (e.g. N-(trifluoroacetyl)-14-phenyl-14-selenaadriamycin). Since ChemSpider strives to include only valid structures, these names are not expected to be found using ChemSpider. The poor quality of chemical names in common usage was discussed by Brecher [17] already in the year 1999 and is 10 years later still an issue. Although many chemistry journals nowadays have rules about the naming of compounds and demands on the addition of structure information, this information has not always been updated for older issues, and unfortunately few MEDLINE abstracts contain structure information.

Database coverage applies to the following cases: three chemicals were present in the database as structures but lacked the specific synonym used in the abstract, and one was not present at all in the database at the time of this study (8-(methylthio)-1,2,3,4,5,6-hexahydro-2,6-methano-3-benzazocine) but has since been added to the database. These cases therefore fit into the *not in dictionary *category.

The fact that many of the random false negatives were found in the online ChemSpider database put the low recall of the manually curated ChemSpider dictionary in a different light. The ongoing online community-based annotation of chemical names in ChemSpider will ensure an increase in recall of the dictionary while hopefully maintaining the precision, and surely the important link to chemical structure. Chemlist requires a more thorough accuracy check of text-mining results due to the lower precision compared to the ChemSpider dictionary but will retrieve more entities. On the other hand, in contrast to ChemSpider, Chemlist is downloadable in its whole and can be used as a basis for the creation of a manually curated chemical dictionary for text mining. Structure information can be added for the entities lacking this information once it is available in the underlying databases.

Partial matches of compounds were an important issue for both dictionaries and something that might be solved by detection of chemical name boundaries before matching. However, the false positives in this category did not decrease when a system that uses this type of information (OSCAR3, available at http://sourceforge.net/projects/oscar3-chem) was tested [13] and further testing of different algorithms for chemical name boundary detection in combination with dictionary look-up is needed.

## Conclusions

We conclude the following: (1) The Chemlist dictionary had the highest recall (0.40) and the best F-score (0.50), but a lower precision (0.67) than the ChemSpider dictionary; the ChemSpider dictionary achieved the best precision (0.87) but at a cost of lower recall (0.19) than the Chemlist dictionary; It should be noted that the ChemSpider dictionary of ca. 80 k names was only a 1/3 to a 1/4 the size of Chemlist at around 300 k and this would be expected to dramatically impact recall. (2) Rule-based filtering and disambiguation is necessary to achieve a high precision for both the automatically generated and the manually curated dictionary.

### Experimental

#### Dictionary pre-processing

The combined chemical dictionary Chemlist has been described elsewhere [13]. Briefly, it is based on the following resources: the chemical part of the Unified Medical Language System metathesaurus (UMLS) [8], the chemical part of the Medical Subject Headings (MeSH) [18], the ChEBI ontology [19], DrugBank [7], KEGG drug [20], KEGG compound [21], the human metabolome database (HMDB) [22], and ChemIDplus [23]. Data from the fields used for entry term, synonyms, summary structure, and database identifiers were used to build the Chemlist dictionary. CAS registry numbers [24] and Beilstein reference numbers [25] were not used for text mining due to their presumed ambiguity with other number types in text. CAS numbers do have a specific format that should help identify them in text and might be included as synonyms in future releases of the dictionary. Entries were merged if they had the same CAS number, database identifier (cross-reference), or InChI string. No manual curation was performed to ensure the correctness of merged entities. A set of rules was used to rewrite and suppress terms in the dictionary and a manual check for highly frequent terms was performed [13]. Briefly, we *removed *a term if (1) the whole term after tokenization and removal of stop words is a single character, or is an arabic or roman number (e.g. "T" as an abbreviation for "Tritium"); (2) the term contained any of the following features: a dosage in percent, gram, microgram or milliliter, "not otherwise specified", "not specified", or "unspecified", "NOS" at the end of a term and preceded by a comma, or "NOS" within parentheses or brackets at the end of a term and preceded by a space, "other" at the beginning of a term and followed by a space character or at the end of a term and preceded by a space character, "deprecated", "unknown", "obsolete", "miscellaneous", or "no" at the beginning of a term and followed by a space character (e.g. "unspecified phosphate of chloroquine diphosphate" as synonym for "chloroquine diphosphate"); (3) the term corresponded to a general English term in the top 500 most frequent terms found in a set of 100,000 randomly selected MEDLINE abstracts indexed with the Chemlist dictionary. We *added *(1) the syntactic inversion (e.g. "acid, gamma-vinyl-gamma-aminobutyric" is rewritten to "gamma-vinyl-gamma-aminobutyric acid"); (2) the stripped possessive version (e.g. "Ringer's lactate" rewritten to "Ringer lactate"); (3) the long form and short form version of a term (e.g. "Hydrogen chloride (HCL)" is split into "Hydrogen chloride" and "HCL"). Since a rewritten term will be added to the dictionary without removing the original term, an increase in synonyms after using the rewrite rules will take place.

We acquired a dictionary subset from the chemical database ChemSpider (February 12, 2009), containing only manually annotated names and synonyms. Before manual curation, robots had been used to ensure that there were no inappropriate correspondences between the chemical names and the chemical structures. For example, it is rather common in the public databases to have the chemical names of salts despite the fact that the chemical itself may be a neutral compound. A series of processing runs to clean up mis-associations in the following manner improved the validity of names associated with structures: 1) for names containing chloride, bromide, iodide, and fluoride check the molecular formulae for the presence of the associated halogens in the molecular formula and treat as necessary; 2) for names containing nitrite, nitrate, sulfate/sulphate, and sulfite/sulphite, check molecular formulae for presence of nitrogen or sulphur and remove names as necessary; 3) for hydrate/dihydrate, check for presence of one or more waters of hydration and remove names as appropriate; 4) convert names to chemical structures using commercial software tools and check for consistency and flag as checked by robots. This is a different level of curation than checked by humans. The manually annotated names are those approved primarily by users of ChemSpider and then further validated by master curators. The result is a highly curated database of chemical structures with their associated manually curated identifiers. These identifiers are not limited to systematic names and trade names but also include CAS registry numbers, EINECS or ELINCS numbers [26] and Beilstein reference numbers. In order to make a fair comparison with the Chemlist dictionary, we applied the same filtering rules and manual check for highly frequent terms to the ChemSpider dictionary as were previously applied to the Chemlist dictionary. This time, the manual check for highly frequent terms was based on a MEDLINE indexation using the ChemSpider dictionary.

#### Term identification

We used our concept recognition software Peregrine [27] to index a corpus of annotated chemical abstracts from Kolarik et al. [16]http://www.scai.fraunhofer.de/chem-corpora.html. The Peregrine system translates the terms in the dictionary into sequences of tokens. When such a sequence of tokens is found in a document, the term, and thus the chemical associated with that term, is recognized. Some tokens are ignored, since these are considered to be non-informative ('of', 'the', 'and', 'in'). The tokenizer in Peregrine considers everything that is not a letter or a digit to be a word delimiter. Similar to Hettne et al. [13], we made the following adjustments to the tokenizer: full stops, commas, plus signs, hyphens, single quotation marks and all types of parentheses ((, {, [) were excluded from the word delimiter list. After tokenization, the tokens were stripped of trailing full stops, commas and non-matching parentheses. Parentheses were also removed if they surrounded the whole token. In addition, a list of common suffixes was used to remove these suffixes at the end of tokens [13]. We used Peregrine with the following settings: case-insensitive, word-order sensitive and largest match.

The annotated corpus consists of 100 MEDLINE abstracts with 1206 annotated chemical occurrences divided into the following groups: multiword systematic names (IUPAC, 391 occurrences), partial chemical names (PART, 92 occurrences), sum formulas (SUM, 49 occurrences), trivial names (including single word IUPAC names) (TRIV, 414 occurrences), abbreviations (ABB, 161 occurrences), and chemical family names (FAM, 99 occurrences). Larger drug molecules such as protein drugs had not been annotated in the corpus [16]. The creators used a simple system for detecting IUPAC names [28] to select abstracts containing at least one found entity. Next to abstracts selected with this procedure, they selected abstracts containing problematical cases as well as abstracts containing no entities. The inter-annotator F1 was 80% when recognizing the boundaries without considering the different classes.

We indexed the corpus using three versions of the ChemSpider dictionary: unprocessed, filtered (after application of the filtering rules), and frequent terms correction (after the check for frequent English terms). To compare the effect of disambiguation rules during the indexing process we used the same rules as in Hettne et al. [13]. That is, we first determine whether a term is a dictionary homonym, i.e., if it refers to more than one entity in the dictionary. If the term is a dictionary homonym, but it is the preferred term of that entity, it is further handled as if it is not a dictionary homonym. If the term is not a dictionary homonym it still needs further processing since it can have many meanings in text. Therefore, terms that are shorter than five characters or do not contain a number are also considered potential homonyms, and require extra information to be assigned. A (potential) homonym is only kept if (1) another synonym of the entity is found in the same piece of text; (2) a keyword (i.e., a word or "token" that occurs in any of the long-form names of the small molecule, and appears less than 1000 times in the dictionary as a whole) is found in the same piece of text. The results from the ChemSpider dictionary were compared to the results previously reported for the Chemlist dictionary.

#### Error analysis

A random set of maximum 25 false negatives from each class of entities in the corpus, the 232 false positives for Chemlist, and the 33 false positives for the ChemSpider dictionary were analyzed. For comparison, we used the same error categories for the false negatives and false positives as in Hettne et al. [13]. For the false negatives, these were: *partial match *(e.g. only "beta-cyclodextrin" in "hydroxypropyl beta-cyclodextrin" was recognized); *annotation error *(e.g. only part of the chemical name has been marked by the annotators in the text: "thiophen" in the sentence"... Gewald *thiophen***e **synthesis was...", or a whole entity has been overlooked by the annotators); *not in dictionary*; *removed by disambiguation *(e.g. single letter "T"); *removed by manual check of highly frequent terms *(e.g. "Me"); and *tokenization error *(e.g. "Ca(2+)" will not be found in the sentence "...free calcium concentration ([Ca(2+)]i) of human peripheral blood lymphocytes..." due to the positioning of the "i" that does not allow the surrounding brackets to be removed from the entity). The error categories for the false positives were: *partial match*; *annotation error*; *out of corpus scope *(e.g. larger drug molecules such as protein drugs); *not a chemical *(e.g. the term "metabolite").

## Competing interests

The authors declare that they have no competing interests.

## Authors' contributions

KMH participated in the design of the study, performed the data analysis and drafted the manuscript. AJW participated in the design of the study, participated in the data analysis and helped to draft the manuscript. EMM participated in the design of the study and helped to draft the manuscript. JK helped to draft the manuscript. VT participated in provision of data and helped to draft the manuscript. JAK participated in the design of the study and helped to draft the manuscript. All authors read and approved the final manuscript.
