# Automatic vs. manual curation of a multi-source chemical dictionary: the impact on text mining

**DOI:** 10.1186/1758-2946-2-4

**Published:** 2010-06-03

**Authors:** Kristina M Hettne, Antony J Williams, Erik M van Mulligen, Jos Kleinjans, Valery Tkachenko, Jan A Kors

**Affiliations:** 1 Department of Medical Informatics, Erasmus University Medical Center, Rotterdam, The Netherlands; 2 Department of Health Risk Analysis and Toxicology, Maastricht University, Maastricht, The Netherlands; 3 Royal Society of Chemistry, 904 Tamaras Circle, Wake Forest, NC-27587, USA

## Correction

In 'Automatic vs. manual curation of a multi-source chemical dictionary: the impact on text mining' (Hettne et al. Journal of Cheminformatics 2010, 2:3) [1], the name of the automatically curated dictionary is identified as 'Chemlist'. CHEMLIST is a trademark that the American Chemical Society has used for many years to identify its Regulated Chemicals Listing (CAS) database. To avoid future confusion, the 'Chemlist' dictionary mentioned in this article has been renamed to 'Jochem.'

## References

[B1] Hettne KM Williams AJ van Mulligen EM Kleinjans J Tkachenko V Kors JA Automatic vs. manual curation of a multi-source chemical dictionary: the impact on text mining J Cheminform 2010 2 3 10.1186/1758-2946-2-3 20331846 PMC2848622

